# Use of a Lateral Sinus Bony Window as an Intraoral Donor Site for Guided Bone Regeneration in Wide Post-Extraction Defects

**DOI:** 10.3390/medicina58121785

**Published:** 2022-12-04

**Authors:** Won-Bae Park, Philip Kang, Wonhee Park, Ji-Young Han

**Affiliations:** 1 Department of Periodontology, School of Dentistry, Kyung Hee University, Seoul 02447, Republic of Korea; 2 Private Practice in Periodontics and Implant Dentistry, Seoul 02771, Republic of Korea; 3 Division of Periodontics, Section of Oral, Diagnostic and Rehabilitation Sciences, College of Dental Medicine, Columbia University, New York, NY 10032, USA; 4 Department of Prosthodontics, Division of Dentistry, College of Medicine, Hanyang University, Seoul 04763, Republic of Korea; 5 Department of Periodontology, Division of Dentistry, College of Medicine, Hanyang University, Seoul 04763, Republic of Korea

**Keywords:** autografts, dental implant, intraoral donor, bone transplantation, lateral sinus window, sinus floor augmentation

## Abstract

Maxillary sinus augmentation (MSA) and guided bone regeneration (GBR) have shown successful clinical, radiological, and histological outcomes for implant-related bone reconstruction and have been used to augment bony defects of various shapes and sizes. This study demonstrated that the lateral sinus bony window obtained during MSA can be used as an autogenous block bone graft for the augmentation of wide post-extraction defects. During the uncovering procedure performed 6 months after surgery, the grafted lateral bony window was well integrated with the adjacent native bone, and complete bone filling was observed in all bony defects around the implants. All of the implants survived. Within the limitations of this study, autogenous block bone obtained from lateral window sites can be used as novel donors for the resolution of wide bony defects around implants.

## 1. Introduction

Guided bone regeneration (GBR) has been clinically applied for over 40 years and has shown successful clinical, radiological, and histological outcomes. In particular, GBR procedures have been widely used in osseous defects around implants and have enabled the long-term survivability of implants [1,2]. In line with this concept, various types of bone graft substitutes have been developed for the resolution of defects around implants, and various membranes have been used for cell occlusive and promotive osteogenesis [2,3].

Autogenous bone is considered the gold standard among bone graft substitutes that can be used for osseous defects [4]. Autogenous bone contains vital cells with osteogenic potential [5]. However, it has the disadvantages of donor morbidity and a limited amount for use. Among the nonautogenous bone grafts, such as allograft, xenograft, and synthetic bone grafts, particulate bone has limitations for maintaining ridge contours in wide defects. On the other hand, autogenous block bone can maintain the desired shape, which is advantageous for the reconstruction of wide defects [6].

The ascending ramus, symphysis area, mandibular tori, maxillary tuberosity, and zygomatic alveolar crest have been used as intraoral donor sites of autogenous bone [7]. Recently, it was reported that the obtained core bone from a previously augmented sinus was used as a bone graft substitute [8].

In the lateral window technique for maxillary sinus augmentation (MSA), various methods for managing the lateral sinus bony window have been introduced [9,10]. Zuzikis et al. outlined four options for lateral sinus bony windows in a literature review: the bony window is elevated into the sinus cavity under the sinus membrane; removed and discarded; crushed and mixed with other particulate bone graft substitute for MSA; and repositioned to its original position after MSA [11]. Among these methods, lateral repositioning is a clinically and histologically effective procedure [12,13]. As a result, the repositioned window was introduced as a good alternative to membrane placement [9,14]. In addition, due to the osteogenic potential of lateral sinus window bone, it can be used as an autogenous bone graft when implant placement is performed simultaneously as sinus augmentation in severe post-extraction defects. Therefore, we used lateral sinus window bone for the augmentation of bony defects around implants. To the best of the authors’ knowledge, few reports have introduced the lateral sinus bony window obtained during MSA as an intraoral donor for GBR in wide osseous defects.

In the present case report, we aimed to show a GBR procedure for wide osseous defects using the lateral sinus bony window obtained during a lateral MSA.

## 2. Case Descriptions

Four patients who underwent GBR procedures using the lateral sinus bony window as an intraoral donor for wide osseous defects were included in this case report. The demographic characteristics of the patients, sites, and implants are shown in Table 1.

### 2.1. Case 1

**Figure 1:**
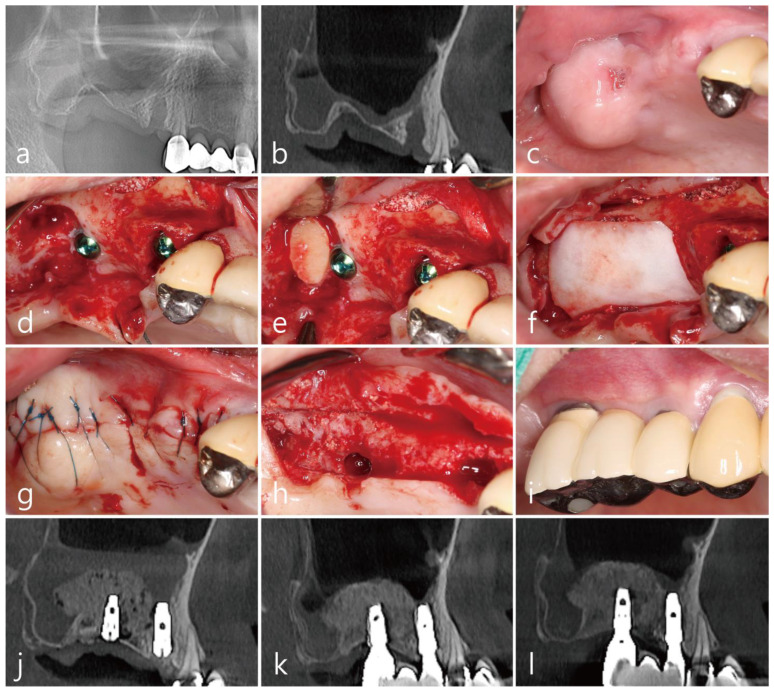
Case 1: (**a**) preoperative panoramic radiograph; (**b**) sagittal image of the preoperative CBCT scan showing a severely resorbed bone defect; (**c**) preoperative clinical view two months after tooth extraction; (**d**) after the flap reflection, a lateral window was prepared, and the buccal bony window was obtained during the maxillary sinus augmentation; (**e**) the obtained lateral bony window was passively placed on the distal intraosseous defect of the #17 implant without fixation; (**f**) the surgical site was covered with a resorbable collagen membrane; (**g**) the flaps were closed with minimal tension; (**h**) the uncovering procedure was performed six months after the operation, and the lateral window bone was well incorporated with the adjacent native bone; (**i**) clinical view at two years after prosthesis delivery; (**j**) panoramic radiograph immediately after surgery; (**k**) sagittal image of the CBCT scans after prosthesis delivery; (**l**) sagittal image of the CBCT two years after prosthesis delivery, where the lateral bony window was well incorporated with the surrounding native bone, and the bone density was increased.

The patient was a 72 years old nonsmoking female, who visited the clinic for implant placement in the right posterior region of the maxilla (Table 1). She had undergone extractions of the maxillary right second premolar and second molar two months prior. The extraction site of the maxillary second molar exhibited severe vertical bone resorption. In addition, the right maxillary sinus was severely pneumatized (Figure 1a). The sagittal image of the preoperative cone-beam computed tomography (CBCT; Rainbow^TM^ CT, Dentium, Suwon, Republic of Korea; exposure time 10 s) scan revealed an intraosseous defect in the extraction site of the maxillary right second molar (Figure 1b). No posterior superior alveolar artery (PSAA) in the lateral sinus wall was observed in the CBCT scans (Table 1). A vertical deficiency of the soft tissue was also observed (Figure 1c). Lateral MSA and simultaneous implant placement were planned. The flaps were reflected under local anesthesia with 2% lidocaine (containing 1:100,000 epinephrine). After the removal of the granulation tissue in the extraction sites, MSA was performed using the lateral window technique with an Osteon II (Genoss Co., Ltd., Suwon, Republic of Korea), and the lateral sinus bony window was obtained. Two SLA-textured implants (Ø4.3 × 12 mm and Ø4.8 × 10 mm; Implantium, Dentium Co., Ltd., Suwon, Republic of Korea) were placed (Table 1, Figure 1d). The obtained lateral sinus bony window was passively placed without fixation to an intraosseous defect on the distal aspect of the implant placed in the right maxillary secondary molar site (Figure 1e). The surgical site was subsequently covered with a resorbable collagen membrane (Genoss Co., Ltd., Suwon, Republic of Korea) (Figure 1f), and the flaps were closed with 5-0 nylon sutures (Figure 1g). Antibiotics (ciprofloxacin 500 mg; Ildong Pharmaceutical Co., Ltd., Seoul, Republic of Korea) and a nonsteroidal anti-inflammatory drug (Etodol; 200 mg; Yuhan Co., Ltd., Seoul, Republic of Korea) were prescribed for 10 days. The sutures were removed after two weeks. Transient post-operative swelling and pain were reported, but healing was uneventful. The uncovering procedure was performed six months after the surgery (Figure 1h). A prosthesis was delivered two months after the uncovering procedure. Two years after prosthesis delivery, no abnormal findings were observed around the implant (Figure 1i). The sagittal view of the CBCT scan performed immediately after surgery shows a well-placed lateral window bone in the distal defect of the implant placed in the maxillary right second molar site (Figure 1j). In the sagittal image of the CBCT scan performed immediately after the prosthesis delivery, the lateral sinus window bone was well incorporated with the surrounding native bone (Figure 1k). The sagittal CBCT scans two years after the prosthesis delivery revealed an improved bony density (Figure 1l).

### 2.2. Case 2

**Figure 2:**
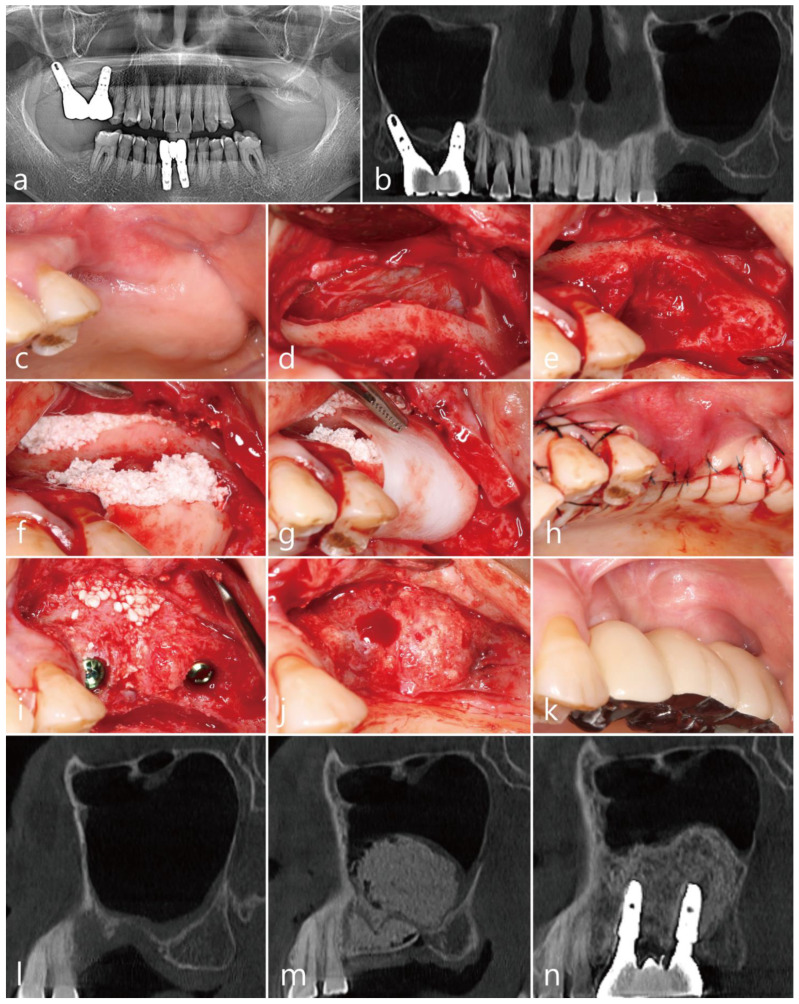
Case 2: (**a**) preoperative panoramic radiograph; (**b**) preoperative panoramic image of the CBCT scan showing a severely resorbed bone defect and a pneumatized maxillary sinus on the left side; (**c**) preoperative clinical view at two months after extraction of the left maxillary second premolar and first molar; (**d**) after reflection of the mucoperiosteal flap, the lateral bony window was obtained; (**e**) thorough debridement was performed to remove inflammatory granulation tissue; (**f**) after maxillary sinus augmentation, an appropriately trimmed lateral bony window was placed on the defect, and the gap was filled with particulate bone graft; (**g**) the grafted site was covered with a collagen membrane; (**h**) the mucoperiosteal flaps were closed with minimal tension; (**i**) implants were placed six months after the surgery, and additional bone grafting was performed at the lateral window site where bone loss had occurred due to the sinus graft infection; (**j**) the uncovering procedure was performed six months after implant placement; (**k**) a prosthesis was delivered two months after the uncovering procedure; (**l**) preoperative sagittal image of the CBCT scan; (**m**) sagittal images of the CBCT taken immediately after surgery; (**n**) sagittal image of the CBCT one year after prosthesis delivery.

The patient was a 78 year old nonsmoking woman, who visited the clinic for implant placement in the left maxillary posterior region (Table 1). The patient was taking antihypertensive drugs and had no other diseases that could interfere with the surgery. Two months after the extractions of #25 and #26, panoramic radiographs and CBCT scans showed severe bone deficiencies in the extraction sockets (Figure 2a,b). There was no PSAA in the lateral sinus wall (Table 1). The preoperative clinical photographs revealed severe deficiencies of the hard and soft tissues of the extraction sites (Figure 2c). Under local anesthesia with 2% lidocaine (containing 1:100,000 epinephrine), the mucoperiosteal flaps were reflected, and the lateral bony window obtained during MSA was removed (Figure 2d). There was no perforation of the maxillary sinus mucosa during MSA. The extraction socket had a severe bone defect. Thorough debridement was performed to remove inflammatory granulation tissue (Figure 2e). After the MSA procedure was performed, the post-extraction defect was augmented using a particulate bone graft substitute (Osteon™ III; particle size 0.2–0.5 mm; Genoss Co., Ltd., Suwon, Republic of Korea) was performed. Then, a lateral window bone was appropriately trimmed using a grinder (Dask kit, Dentium Co., Ltd., Suwon, Republic of Korea) and was placed in the extraction defects of #25 and #26 (Figure 2f). The bone graft site was covered with a resorbable collagen membrane (Genoss Co., Ltd., Suwon, Republic of Korea) (Figure 2g), and the flaps were closed with 5-0 sutures (Figure 2h). Antibiotics (ciprofloxacin; 500 mg; Ildong Pharmaceutical Co., Ltd., Seoul, Republic of Korea) and a nonsteroidal anti-inflammatory drug (Etodol; 200 mg, Yuhan Co., Ltd., Seoul, Republic of Korea) were prescribed for two weeks. A total of 0.12% of chlorhexidine (hexamedine; Bukwang Pharmaceutical, Seoul, Republic of Korea) mouth rinsing was recommended twice a day for two weeks. Two weeks after surgery, a sinus graft infection occurred, and incision and drainage were performed at the lateral window site. An additional antibiotic (ciprofloxacin; 500 mg; Ildong Pharmaceutical Co., Ltd., Seoul, Republic of Korea) were administered for 7 days. Saline irrigation was performed once a week for six weeks. The implant was placed six months after the MSA. Due to the sinus graft infection, some resorption occurred in the grafted area of the lateral sinus window, and an additional bone graft was performed (Figure 2i). The uncovering procedure was performed six months after the implant placement. The preoperative bony defects were filled to the top of the cover screw (Figure 2j). A prosthesis was delivered two months after the uncovering procedure (Figure 2k).

The preoperative sagittal CBCT scan revealed severely resorbed extraction sockets of #25 and #26 (Figure 2l). On the sagittal image of the CBCT immediately after surgery, the lateral window bone obtained during MSA was located at the entrance of the extraction socket (Figure 2m). The sagittal CBCT scan one year after the prosthesis delivery revealed homogeneous bone filling compared to the previous CBCT scans (Figure 2n).

### 2.3. Case 3

**Figure 3:**
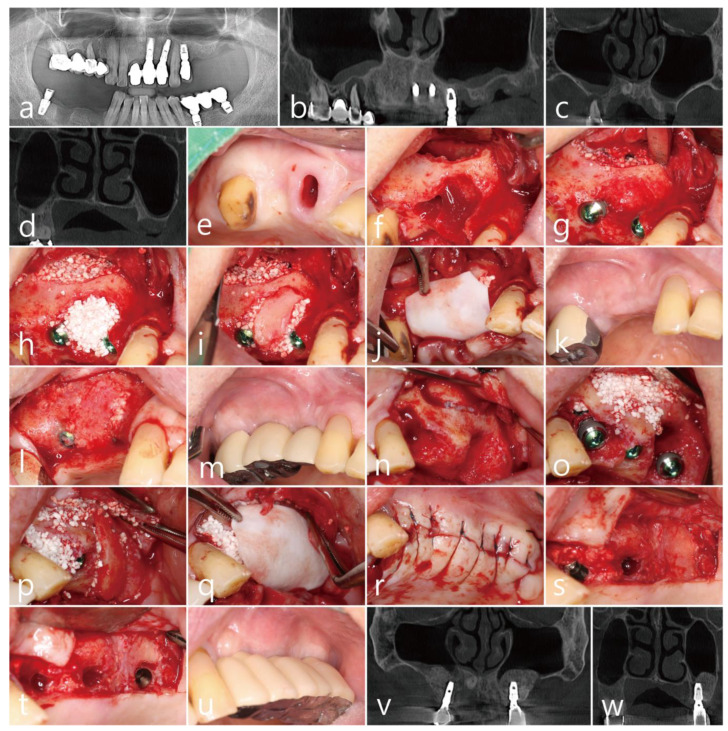
Case 3: (**a**) preoperative panoramic radiograph; (**b**) preoperative panoramic image of the CBCT scan; (**c**) posterior superior alveolar arteries (PSAAs) are shown on the coronal images of the preoperative CBCT scans on both sides; (**d**) coronal image of the preoperative CBCT scan showing a severely resorbed bone defect around the extraction site of the left maxillary second molar; (**e**) preoperative clinical view after extraction of the right maxillary second premolar; (**f**) after flap reflection, a lateral bony window was prepared for maxillary sinus augmentation; (**g**) a bony defect was observed after implant placement and sinus augmentation; (**h**) the buccal bony defect was filled with particulate bone graft; (**i**) the obtained lateral bony window was placed on the bony defect without fixation; (**j**) the surgical site was covered with a resorbable collagen membrane; (**k**) clinical view six months after surgery; (**l**) the lateral bony window was well incorporated with the surrounding native bone at six months after surgery; (**m**) clinical view after final prosthesis delivery; (**n**) after flap reflection, the #24 implant was explanted and a lateral bony window was prepared for maxillary sinus augmentation; (**o**) after sinus augmentation and implant placement, the buccal bony defects are shown; (**p**) after filling the buccal bony defect with particulate bone graft, the obtained lateral bony window was positioned on the bony defect; (**q**) the surgical site was covered with a resorbable collagen membrane; (**r**) the flaps were closed with minimal tension; (**s**) the uncovering procedure was performed six months after surgery, and the grafted lateral bony window was well integrated with the surrounding native bone; (**t**) the bone above the cover screw was removed with a round surgical bur, and after the healing abutments were inserted, the flaps were closed; (**u**) clinical view after final prosthesis delivery; (**v**) coronal CBCT image at six months after prosthesis delivery; (**w**) coronal CBCT scan showing the filling of the defect around the implant placed at the extraction site of the left maxillary second molar.

The patient was a 65 years old nonsmoking male, who visited the clinic for the treatment of a failed implant and re-implantation. The patient did not have any systemic diseases that could interfere with the operation, except for hyperlipidemia. A preoperative panoramic radiograph revealed severely resorbed bone around #15 (Figure 3a). On the panoramic image of the CBCT before the surgery, pneumatization of the left maxillary sinus was observed. In addition, severe bone resorption due to the fact of peri-implantitis was observed on the distal aspect of the #24 implant (Figure 3b). On the preoperative coronal CBCT scan, the buccal bone of the #15 tooth was severely resorbed (Figure 3c), and the residual bone height of the left posterior molar site was significantly reduced (Figure 3d). PSAAs were observed on both lateral sinus walls. On the right, the diameter of the PSAA was 1.5 mm, and the vertical position was 7.3 mm from the floor of the right maxillary sinus. On the left, the diameter of the PSAA was 1.3 mm, and the vertical position was 11.2 mm from the floor of the left maxillary sinus (Table 1).

After removal of the old prosthesis, the #15 tooth was extracted under local anesthesia with 2% lidocaine (containing 1:100,000 epinephrine) (Figure 3e). The mucoperiosteal flaps were reflected, followed by a lateral window preparation for MSA. The lateral sinus bony window was obtained, and the sinus membrane detached from the sinus floor without perforation. The severe bone defect around the extraction socket of the #15 tooth was observed (Figure 3f). Sinus bone augmentation was performed using an Osteon III (particle size 0.5–1.0 mm; Genoss Co., Ltd., Suwon, Republic of Korea), and implants (Implantium, Dentium Co., Ltd., Suwon, Republic of Korea) were placed. A mesial bony defect was observed around the implant during the procedure (Figure 3g) and was filled with Osteon III (particle size 0.2–0.5 mm; Genoss Co., Ltd., Suwon, Republic of Korea) (Figure 3h). The obtained lateral sinus bony window was placed on the bony defect without fixation after slight trimming to match the size and shape of the defect (Figure 3i). The surgical site was covered with a resorbable collagen membrane (Genoss Co., Ltd., Suwon, Republic of Korea) (Figure 3j). The mucoperiosteal flaps were closed with 5-0 nylon. The healing was uneventful, and the wound was not exposed (Figure 3k). The uncovering procedure was performed six months after the surgery. The grafted lateral window bone was well integrated with the surrounding bone (Figure 3l). Healing abutments were inserted, and the flaps were closed. After two months, the final prosthesis was delivered (Figure 3m).

On the same day, lateral MSA and implant placement were performed in the left maxillary posterior region. Under local anesthesia with 2% lidocaine (containing 1:100,000 epinephrine), the mucoperiosteal flaps were reflected. The implant placed in the #24 site was explanted, and a lateral sinus window site was prepared for MSA. The extraction socket of the #27 tooth had a wide-deep defect (Figure 3n). The MSA using an Osteon III (particle size 0.5–1.0 mm; Genoss Co., Ltd., Suwon, Republic of Korea) was performed, and three implants were placed (Table 1). A buccal bony defect was observed around the #27 implant site (Figure 3o). After filling the bony defects with Osteon III (particle size 0.2–0.5 mm; Genoss Co., Ltd., Suwon, Republic of Korea), the obtained lateral sinus bony window was positioned in the #27 implant site (Figure 3p). The surgical site was covered with a resorbable collagen membrane (Genoss Co., Ltd., Suwon, Republic of Korea) (Figure 3q), and the flaps were closed with 5-0 nylon (Figure 3r). Antibiotics (ciprofloxacin; 500 mg; Ildong Pharmaceutical Co., Ltd., Seoul, Republic of Korea) and a nonsteroidal anti-inflammatory drug (Etodol; 200 mg; Yuhan Co., Ltd., Seoul, Republic of Korea) were prescribed for two weeks. The healing was uneventful. The uncovering procedure was performed six months after the surgery. The buccal bony defects around the #27 implant were resolved, and the transplanted lateral sinus window was well integrated with the surrounding native bone (Figure 3s). The bone above the cover screw was removed using a round surgical bur (Figure 3t). The prosthesis was delivered after two months (Figure 3u).

The sinus augmented bone obtained from the MSA was well integrated on the coronal images of the CBCT performed six months after the final prosthesis was delivered (Figure 3v,w).

### 2.4. Case 4

**Figure 4:**
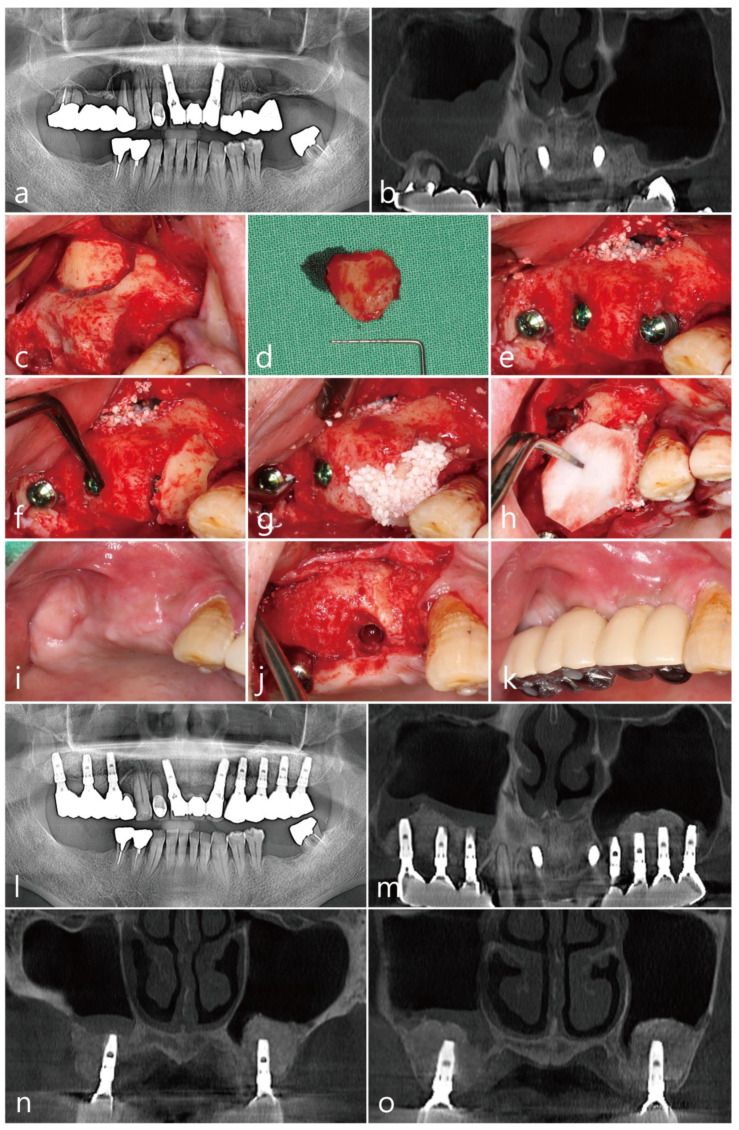
Case 4: (**a**) preoperative panoramic radiograph; (**b**) panoramic image of the preoperative CBCT scan; (**c**) after flap reflection, the lateral bony window was prepared; (**d**) obtained lateral bony window; (**e**) implants were placed after the maxillary sinus augmentation; (**f**) the lateral bony window was cautiously trimmed and placed on the mesial bony defect; (**g**) additionally, particulate bone graft was used to fill the gap; (**h**) a resorbable collagen membrane was placed on the grafted site; (**i**) a clinical view at six months after surgery; (**j**) the grafted lateral bony window was well integrated with the surrounding native bone; (**k**) clinical view after prosthesis delivery; (**l**) panoramic radiograph at six months after prosthesis delivery; (**m**) panoramic image of the CBCT at six months after prosthesis delivery; (**n**) coronal image of the CBCT scan showing posterior superior alveolar arteries on both sides; (**o**) coronal image of the CBCT scan showing reduced mucosal thickening on the right maxillary sinus.

The patient was a 72 year old nonsmoking male (Table 1). An old prosthesis in the maxillary right posterior area was severely mobile, and the patient experienced discomfort during mastication. The patient requested implant-supported restoration after the removal of the old prosthesis. The preoperative panoramic radiograph showed severe bone resorption around teeth #14 and #17. A panoramic image of the CBCT before the surgery showed bony defects around the teeth and severe sinus mucosal thickening (Figure 4a,b).

Tooth extraction, MSA, and implant placement were planned simultaneously. Under local anesthesia with 2% lidocaine (containing 1:100,000 epinephrine), the mucoperiosteal flaps were reflected after extractions of the #14 and #17 teeth in the right maxillary posterior region. The lateral sinus window site was prepared using a #6 round bur (Ø1.6 mm; Azdent, Zhengzhou, Henan Province, China) (Figure 4c). The lateral bony window was obtained (Figure 4d). The sinus membrane was detached and elevated using a sinus elevation instrument (Genoss Co., Ltd., Suwon, Republic of Korea) without perforation. Three implants (Implantium, Dentium Co., Ltd., Suwon, Republic of Korea) were placed after MSA using an Osteon III (particle size 0.5–1.0 mm; Genoss Co., Ltd., Suwon, Republic of Korea) (Table 1). A severe bony defect was observed at the mesial site of the implant placed in the #15 site (Figure 4e). The obtained lateral sinus bony window was cautiously trimmed and placed in the mesial bony defect (Figure 4f). In the residual defect around the grafted lateral sinus bony window, a particulate bone graft substitute (Osteon III; particle size 0.2–0.5 mm; Genoss Co., Ltd., Suwon, Republic of Korea) was grafted (Figure 4g). The wound was covered with a resorbable collagen membrane (Genoss Co., Ltd., Suwon, Republic of Korea) (Figure 4h) and closed with 5-0 nylon. Antibiotics (ciprofloxacin; 500 mg; Ildong Pharmaceutical Co., Ltd., Seoul, Republic of Korea) and a nonsteroidal anti-inflammatory drug (Etodol; 200 mg; Yuhan Co., Ltd., Seoul, Republic of Korea) were prescribed for two weeks. No complications occurred during the healing, and the wound was not exposed (Figure 4i). The uncovering was performed six months after the surgery. The mesial bony defect around implant #15 was completely resolved, and the grafted lateral sinus bony window was well incorporated with the surrounding native bone (Figure 4j). The final prosthesis was delivered two months after the uncovering procedure (Figure 4k).

On the panoramic radiograph after the prosthesis delivery, the mesial bony defect of the #15 implant was filled with the grafted lateral sinus bony window and particulate bone graft (Figure 4l). The panoramic image of the CBCT scan showed a reduced sinus mucosal thickening and the incorporation of the grafted lateral sinus bony window with the surrounding native bone (Figure 4m). The coronal CBCT scans showed PSAAs on both sides. The diameter of the PSAA was 1.1 mm, and the vertical position from the floor of the right maxillary sinus was 19.9 mm (Table 1). On the coronal image of the CBCT at the #15 implant, the mesial bony defect was well resolved (Figure 4n). The coronal CBCT scan at the #16 implant (Figure 4o) showed reduced sinus mucosal thickening compared to the preoperative CBCT scan (Figure 4b).

## 3. Discussion

The clinical, radiological, and histological outcomes of the GBR technique using autogenous bone for the reconstruction of atrophied alveolar ridges, MSA, and bony defects around implants have been verified through many studies and clinical applications [15,16,17]. In general, the lateral window technique of the maxillary sinus is used for MSA, but the present case series showed that the obtained lateral sinus bony window can be used for augmentation of wide bony defects adjacent to the lateral augmented sinus. 

Autogenous bone grafting is considered a gold standard among bone graft substitutes due to the fact of its osteogenic potential and lack of immunogenicity [17,18]. Currently, autogenous bone grafts have been widely replaced with bovine, porcine, and equine xenografts [17,18]. In the cases of small, localized bone deficiencies, such as extraction sockets and thin alveolar ridges, the choice of bone graft substitute is not a critical factor for successful outcomes [19]. However, in cases of extensive bone deficiencies or ridge discontinuity defects, there is nothing better than an autogenous bone graft. In the present case report, we used lateral sinus window bone for the augmentation of bony defects around implants. The most common complication for autogenous block bone is wound exposure and sequestration of the grafted bone, which were not observed in this study. In cases 3 and 4, immediate implant placement was performed to restore the patient’s chewing function more quickly. The healing period could be shortened due to the osteogenic potential of lateral sinus window bone.

There are several anatomical structures that must be considered when using the lateral wall of the maxillary sinus as a bone graft substitute. The presence of the posterior superior alveolar artery (PSAA) running within the buccal bone of the maxillary sinus is challenging for clinicians. If the diameter of the PSAA is more than 2 mm and overlaps with the lateral window site, careful attention is required during the preparation of the lateral sinus window. The presence of a PSAA is confirmed with an average frequency of 68.2% and 78.12% in CT and CBCT scans, respectively [20,21]. The thickness of the PSAA varies less than 1 to 1.3 mm [20,22]. In these cases, a PSAA was present in two of four patients, and the diameter was from 1.1 to 1.5 mm. The vertical distance from the sinus floor to the PSAA varied from 7.3 to 19.9 mm. In this study, the diameter of the PSAAs was less than 1.5 mm, and its location was higher than that of the lateral window site. Therefore, there was no severe bleeding during the lateral window preparation. Moreover, there have been very few cases of delayed or complicated surgery due to the fact of bleeding from PSAA during lateral window preparations and no reported life-threatening hemorrhage [23].

Another anatomical structure to be considered in sinus augmentation is the sinus septum connected to the sinus floor [24]. When the lateral sinus bony window is detached from the sinus membrane, there is a high possibility of perforation of the Schneiderian membrane [25]. A piezoelectric device has been reported to have a lower rate of membrane perforation compared to a conventional rotating instrument [25]. In this study, no piezoelectric device was used since there was no septum.

Immobilization and vascularization are critical for the success of block bone grafts. If the block bone graft is thick enough, graft immobilization is usually obtained using a fixation screw [5] or the press-fit method [26]. If the thickness of the lateral window bone is greater than 2 mm, it is advantageous for vertical augmentation, and graft fractures can be prevented when a fixation screw is used. A thin lateral window bone can be used for space-making purposes. However, there is a possibility of fracture when a fixation screw is used. The peculiarity of the present study is that when the obtained lateral bony window was placed on the bony defect around the implant, it was passively adapted without using a fixation screw or press-fit method for immobilization. There was no wound exposure or postoperative graft sequestration in these cases. In addition, after six months of healing, the lateral sinus window was well integrated with the surrounding native bone. This was similar to the healing of the lateral bone window repositioned to the lateral window site [13]. We also used particulate bone graft substitutes (particle size 0.2–0.5 mm) for the space between the obtained lateral sinus bony window graft and native bone or implant. This is reported to help blood clot stabilization by reducing the dead space [27].

It is reported that new bone seems to be growing out of the repositioned lateral sinus bony window after MSA [13], and this appears to be due to the osteogenic potential of the autogenous bone graft [13,28]. In the present study, although histologically not yet confirmed, lateral sinus bony windows placed on the bony defects around implants are likely to show similar results to laterally repositioned bony windows after MSA.

A resorbable collagen membrane has been widely used for GBR procedures. The combination of block bone grafts and barrier membranes is intended to minimize the volumetric change by reducing surface resorption during the healing process [29,30]. In the present cases, there was no severe volumetric change after surgery. In case 2, an additional surgical intervention was necessary due to the sinus graft infection after MSA, resulting in greater bone surface resorption compared to the other cases. In the other cases, there were minimal volumetric changes. This study demonstrated for the first time that the lateral sinus bony window can be used for the GBR procedure in a wide post-extraction defect adjacent to the sinus augmented sites. It has been confirmed that the repositioned lateral sinus bony window at the adjacent post-extraction defect incorporates well with the surrounding native bone. This procedure is similar to the repositioning of the lateral window during MSA, which can be performed without coverage of a resorbable collagen membrane [9,10,11]. However, in the defects around implants, the use of a barrier membrane is recommended to reduce surface bone resorption and to prevent exposure of the grafted bone [31,32].

The limitation of this study is that it is difficult to provide clinical guidelines due to the lack of sample size. Nonetheless, the use of lateral sinus bony windows can be an alternative option to intraoral block bone grafts for GBR procedures in wide osseous defects adjacent to sinus augmented sites.

## 4. Conclusions

Within the limitations of the present case series, the lateral sinus bony window obtained during MSA is eligible as a new autogenous intraoral donor that can be used for augmentation of adjacent wide osseous defects.

## Figures and Tables

**Table 1:** Demographic characteristics of the patient, site and implant information, and information of posterior superior alveolar artery and follow-up.

Case	Age	Sex	Smoking	Implant				PSAA		Follow-up (months)
				site	diameter (mm)	length (mm)		Diameter (mm)	Height (mm)	
1	72	Female	No	#15	4.3	12				24
				#17	4.8	10				
2	78	Female	No	#25	4.3	10				12
				#27	4.8	10				
3	65	Male	No	#14	3.8	12		1.5	7.3	6
				#16	4.3	10				
				#24	4.8	10		1.3	11.2	
				#25	4.3	10				
				#27	4.8	10				
4	72	Male	No	#14	3.8	10		1.1	19.9	6
				#15	4.3	10				
				#17	4.3	10				

PSAA: posterior superior alveolar artery.

## Data Availability

Not applicable.
